# 

**Published:** 1904-03-19

**Authors:** 


					the hospital. "The standard of highest purity."?Lancet. . '? march 19th, 1904.
OADBURY'S ma U CADBURY'S
COCOA JL m M, JFS T " COCOA
I "A PERFECT FOOD." 1  ?^ NO DRUOS OR ALKALIES USED.
Ltaoir Wssterh Infi&MAAT
No. 912. Vol. XXXY. [fdr!] Saturday,
March 19th, 1904.
" -mmu^MRwicH
[Eahseription^Tercas,] pR|(JE JHREEpEMQE>

				

